# Biomarker-Guided Assessment of Acute Kidney Injury Phenotypes E among ST-Segment Elevation Myocardial Infarction Patients

**DOI:** 10.3390/jcm11185402

**Published:** 2022-09-14

**Authors:** Ariel Banai, Shir Frydman, Hytham Abu Katash, Moshe Stark, Ilana Goldiner, Shmuel Banai, Yacov Shacham

**Affiliations:** Department of Cardiology, Tel-Aviv Sourasky Medical Center Affiliated to the Sackler Faculty of Medicine, Tel-Aviv University, 6 Weizman St, Tel-Aviv 64239, Israel

**Keywords:** AKI, STEMI, neutrophil gelatinase-associated lipocalin, complications, biomarkers, novel, phenotypes

## Abstract

Recent practice guidelines recommended the use of new stress, functional, and damage biomarkers in clinical practice to prevent and manage acute kidney injury (AKI). Biomarkers are one of the tools used to define various AKI phenotypes and provide prognostic information regardless of an acute decline in renal function. We investigated the incidence and possible implications of AKI phenotypes among ST elevation myocardial infarction patient treated with primary coronary intervention. We included 281 patients with STEMI treated with PCI. Neutrophil gelatinase associated lipocalin (NGAL) was utilized to determine structural renal damage and functional AKI was determined using the KDIGO criteria. Patients were stratified into four AKI phenotypes: no AKI, subclinical AKI, hemodynamic AKI, and severe AKI. Patients were assessed for in-hospital adverse events (MACE). A total of 46 patients (44%) had subclinical AKI, 17 (16%) had hemodynamic AKI, and 42 (40%) had severe AKI. We observed a gradual and significant increase in the occurrence of MACE between the groups being highest among patients with severe AKI (10% vs. 19% vs. 29% vs. 43%; *p* < 0.001). In a multivariable regression model, any AKI phenotype was independently associated with MACE with an odds ratio of 4.15 (95% CI 2.1–8.3, *p* < 0.001,) for subclinical AKI, 4.51 (95% CI 1.61–12.69; *p* = 0.004) for hemodynamic AKI, and 12.9 (95% CI 5.59–30.1, *p* < 0.001) for severe AKI. In conclusion, among STEMI patients, AKI is a heterogeneous condition consisting of distinct phenotypes, addition of novel biomarkers may overcome the limitations of sCr-based AKI definitions to improve AKI phenotyping and direct potential therapies.

## 1. Introduction

Among hospitalized patients, worsening of renal function and acute kidney injury (AKI) is an important complication, associated with adverse outcomes [1,2]. Consensus criteria define AKI based on rise in serum creatinine (sCr), however this often occurs within 48–72 h following renal insult, and after nearly 50% of functional renal mass has been damaged [3]. Under these conditions, therapeutic care bundles are often initiated after glomerular function has already been established and potential irreversible renal damage may already be present. Recent studies have underlined the potential and beneficial role of renal biomarker for early assessment of kidney risk. Various studies examined the use of neutrophil gelatinase-associated lipocalin (NGAL) for the early diagnosis of AKI, demonstrating the accuracy and additional clinical benefits of this biomarker [4,5,6,7,8,9,10,11]. The 23rd Consensus Conference of the Acute Dialysis Quality Initiative (ADQI) suggested the combination of damage and functional biomarkers, along with clinical information, should be used to identify high-risk patient groups, improve the diagnostic accuracy of AKI, improve processes of care, and assist the management of AKI [11]. In accordance with that report, a recent review suggested three AKI phenotypes based on changes in glomerular filtration function and intrinsic structural tubular kidney injury [12]. We aimed to investigate the incidence and clinical relevance of the various AKI phenotypes, utilizing glomerular function and NGAL, as tubular injury marker, for AKI diagnosis, in a cohort of myocardial infarction patients treated percutaneous coronary intervention (PCI).

## 2. Materials and Methods

### 2.1. Patients

We performed a prospective, observational, open label trial in the Tel Aviv Sourasky medical center. We included ST elevation myocardial infarction (STEMI) patients admitted to the cardiac intensive care unit following PCI between November 2017 to May 2022. As plasma NGAL in these patients can reflect chronic inflammation, we excluded patients with chronic inflammatory syndromes and malignancies (*n* = 24). Based on the availability of NGAL kits, the final study population included 281 STEMI patients undergoing PCI. The contrast medium used during PCI was iodixanol (Visipaque, GE Healthcare, Dublin, Ireland). Following PCI, physiologic (0.9%) saline was applied intravenously at a rate of 1 mL/kg/h for 12 h after contrast exposure. In patients with overt heart failure, hydration rate was reduced at the discretion of the attending physician. Echocardiographic examination was performed in all patients within three days following admission to assess left ventricular (LV) ejection fraction. Patient records were evaluated for the occurrence of in adverse events. These included the need for pharmacological/mechanical ionotropic support, development of heart failure episodes (defined as the occurrence of both clinical and radiological signs of congestion) treated conservatively, respiratory failure with the need for mechanical ventilation, cardiac arrhythmias (ventricular fibrillation/tachycardia and new onset atrial fibrillation), bleeding episodes (defined as >3 gr of hemoglobin decrease or the need for blood transfusion), and 30 day mortality. These complications were then combined to form a composite outcome of modified major adverse in-hospital cardiac events (MACE). All cases of AKI were supported by a combined team of intensive care cardiologists and nephrologists who managed the cases. Informed consent was obtained from all individual participants included in the study. Local institutional ethics committee has approved the study protocol (Institutional Board Review: TLV-16-0224).

### 2.2. Laboratory

Venous blood for NGAL analysis were collected within 24 h following admission. Samples were centrifuged within 10 min in a refrigerated centrifuge and stored at −80 °C. Analysis was performed using NGAL rapid Elisa kits (Bioporto Diagnostics, Copenhagen, Denmark). We defined the presence of biomarker related renal damage based on the cardiac surgery associated NGAL score (CSA-NGAL score) with NGAL levels >100 ng/mL suggesting renal damage [13].

The sCr was determined upon hospital admission, prior to PCI, and at least once a day throughout hospitalization, and was available for all analyzed patients. The estimated glomerular filtration rate (eGFR) was estimated using the Chronic Kidney Disease Epidemiology Collaboration (CKD-EPI) equation [14]. Chronic kidney disease (CKD) was categorized as admission eGFR of <60 mL/min/1.73 m^2^ [15]. Functional AKI was determined using the KDIGO criteria [3] and was defined as an increase in sCr ≥ 0.3 mg/dL within 48 h of admission or an increase in sCr ≥ 1.5 times baseline, which was known or presumed to have occurred within the prior seven days. Based on the review by Moledina et al. [12], patients were stratified into four AKI phenotypes based on changes in in NGAL and sCr levels: No damage (NGAL (−)/sCr (−)), subclinical AKI (NGAL (+)/sCr (−)), hemodynamic AKI (NGAL (−)/sCr (+)), and severe AKI with both functional loss and tubular damage (NGAL (+)/sCr (+)).

### 2.3. Statistics

Data are summarized and displayed as mean (± standard deviation) or median (interquartile range 25–75%) for continuous variables and as number (percentage) of patients within each group for categorical variables. The *p* values for continuous variables were determined using the chi square test or Fisher’s exact test when appropriate. We compared two continuous variables were compared using the independent sample *t*-test for normally distributed data and the Mann–Whitney U test for non-normally distributed variables. We applied one-way analysis of variance (ANOVA) for the comparison of four continuous variables. The utilization of AKI phenotypes plasma NGAL levels to assess the risk of any MACE was evaluated by multivariate logistic regression model. Adjusted odds ratio (OR) with 95% confidence intervals (CI) is reported for all variables found significant in the univariate analysis. A two-tailed *p* value of <0.05 was considered significant for all analyses. All the analyses were performed using SPSS 20.0 software (SPSS Inc., Chicago, IL, USA).

## 3. Results

**Figure 1:**
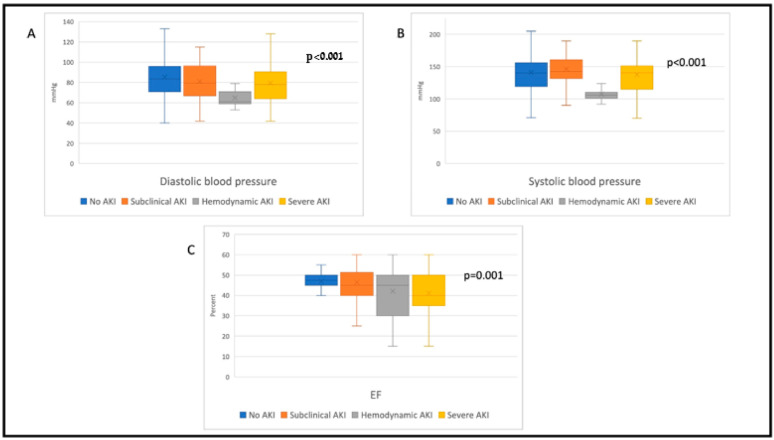
Hemodynamic results by group—(**A**) diastolic blood pressure, (**B**) systolic blood pressure, (**C**) ejection fraction. In all tables, boxplot represents 1st and 3rd quarters, x represents the median, central line represents the mean, extreme brackets represent extreme min max values.

The study population included 281 STEMI patients, mean age was 62 ± 13 years and 78% were men. Overall, 105/281(37%) of patients were diagnosed with any AKI. Of these patients, 46 (44%) had subclinical AKI, 17 (16%) had hemodynamic AKI, and 42 (40%) had severe AKI. Among patients with any AKI, 2 (both having severe AKI) needed transient renal replacement therapy. Patients’ baseline characteristics and laboratory results are shown in Table 1. Patients with hemodynamic AKI had significantly lower systolic and diastolic blood pressures compared with patients with no AKI, subclinical AKI, and severe AKI (*p* < 0.001 for all, Figure 1A,B). In addition, left ventricular ejection fraction was lower among patients with hemodynamic and severe AKI compared to patients with subclinical AKI/no AKI (*p* < 0.001, Figure 1C).

**Figure 2:**
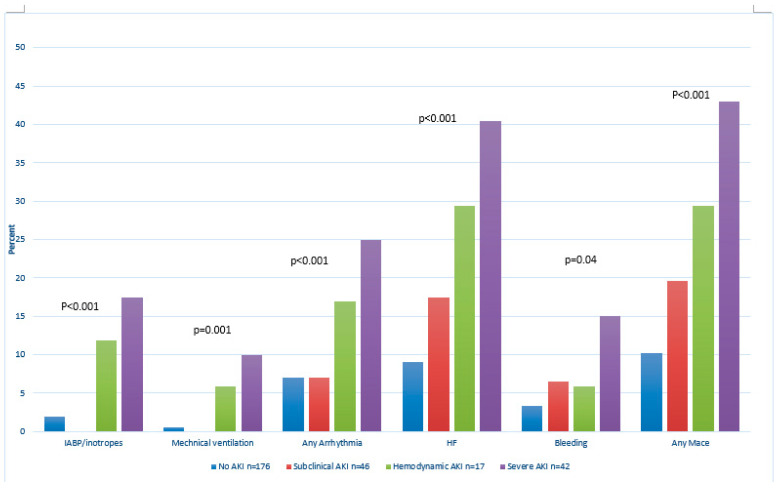
The in-hospital complications according to AKI phenotypes—each complication is presented in a different plot, *p*-value was calculated for trend between the groups. Abbreviations, MACE—major adverse cardiac event.

In-hospital MACE demonstrated a gradual and significant increase between the groups being highest among patients with severe AKI (10% vs. vs 19% vs. 29% vs. 43%; *p* < 0.001, Figure 2). In a multivariable regression model (Table 2), any AKI phenotype was independently associated with MACE with an odds ratio of 4.15 (95% CI 2.1–8.3, *p* < 0.001) for subclinical AKI, 4.51(95% CI 1.61–12.69; *p* = 0.004) for hemodynamic AKI, and 12.9 (95% CI 5.59–30.1, *p* < 0.001) for severe AKI.

## 4. Discussion

This is the first report in a population of STEMI patients, to our knowledge, incorporating contemporary recommendations for clinical adjudication of AKI diagnosis. We demonstrated unique downstream sequelae based on AKI phenotype, with a graded increase in the risk for MACE between patients with subclinical AKI, hemodynamic AKI, and severe AKI.

AKI is common and associated with adverse outcomes [1,2], While imposing a significant health care burden, there currently are no AKI-specific therapies. Although stratification systems to stage AKI by sCr and urine output have been in place for many years [3], there is very limited evidence of published data with regard to the possible effect AKI phenotype on patients’ outcomes. Utilization of routine laboratory parameters may, however, be insufficient under complex and acute conditions to assess AKI. Functional and tubular biomarkers may contribute to the diagnostic and prognostic value of sCr, providing additional pathophysiological information. Such information may be implemented to improve AKI risk-prediction and clinical decision making.

Subclinical AKI is defined as the presence of positive damage biomarker, without an imminent sCr increase [12]. Such positive biomarker test findings were found to be beneficial for outcome and prognosis, regardless of whether renal function acutely declines or not, resulting in tubular injury and loss of glomerular filtration [4,5,6,7,8,9,10,11]. NGAL is induced and released from the injured renal tubules in response to injury [4,5,6]. NGAL’s urine and plasma concentrations increase proportionally to severity and duration of renal injury [7,8], and rapidly decreases with the resolution of renal insult [9]. Thus, increases in NGAL levels predict AKI 24–72 h before diagnostic sCr increases [9,10,11]. Previous reports demonstrated that patients with high NGAL concentrations, without an increase in sCr concentration, required renal replacement therapy more frequently and greater mortality compared with patients without increases in NGAL or sCr concentrations [16,17].

Hemodynamic AKI is defined as a potentially transient decline in eGFR without an obvious structural tubular injury. The pattern and extent of renal tubular injury associated with the hemodynamic AKI phenotype can be distinctly different from other forms of AKI and therefore less biomarker-sensitive [18,19,20]. This may indicate that the worsening of renal function in the hemodynamic AKI phenotype is potentially reversible depending on circulatory adjustment capabilities and physiological /therapeutic volume response. Sustained low cardiac output or severe volume depletion may eventually progress into structural kidney injury and severe AKI. Severe AKI cases more likely result in both structural tubular injury and significant reduction in eGFR with subsequent increase in sCr [21,22,23,24].

Our findings may have important clinical implications. Unfortunately, the distinction between severe AKI and a hemodynamic increase in sCr concentration is made retrospectively based on the clinical course, duration of sCr concentration increase, and response to therapy. Determining the possible prognosis at the time of an AKI event is warranted in order to decide the best management approaches. An important aspect of AKI phenotyping is the ability to prognosticate further progression of an AKI event to higher stage AKI, particularly that requiring renal replacement therapy. In addition, the occurrence of an AKI event is a recognized risk factor for adverse long-term outcomes [25]. AKI is associated with an increased risk of future CKD, end-stage renal disease, cardiovascular morbidity, and mortality [26,27]. Findings at the time of an AKI event may aid in the prediction of these long-term adverse outcomes. Recent reports have also pointed to the possible unique role of NGAL for the prediction of adverse outcomes, regardless of renal injury [28,29]. The ADQI group recently proposed criteria for renal recovery as the absence of AKI by both sCr and urine output criteria within 7 days after AKI onset. For patients in whom pathophysiologic processes are ongoing, the term acute kidney disease (AKD) have been proposed to define the course of disease after AKI [26].

Our study bares notable limitations. This was a single-center study and the number of patients included represents only a modest sample size. We measured only plasma NGAL levels. The addition of urinary NGAL measurement (while not available) could have strengthened the conclusions. NGAL exists in various molecular forms. Monomeric and heterodimeric forms are predominantly secreted by tubular epithelial cells, representing renal injury. The dimeric form originates primarily from granules of matured neutrophils in various inflammatory states. Thus, the elevation of NGAL in STEMI may also reflect the acute inflammatory response to myocardial damage or response to acute decompensated heart failure. We used sCr and eGFR as markers of renal function, knowing that the markers have limitations when used in acute hospitalized patients. Finally, due to the low number of hemodynamic AKI events, a multivariate model to determine predictors of this unique AKI phenotype could have not been performed with accuracy. The usual MACE definition is a composite of cardiac/any death, non-fatal myocardial infarction, repeat revascularization, and stroke. Nevertheless, due to the relatively modest number of patients in the study, we combined the various in-hospital adverse events into the composite end-point of modified MACE.

## 5. Conclusions

Among STEMI patients, AKI is a heterogeneous condition and consist of distinct phenotypes. The addition of novel biomarkers may aid in overcoming limitations of sCr-based AKI definitions improving AKI phenotyping and direct potential therapies.

## Figures and Tables

**Table 1:** Baseline characteristics of 281 STEMI patients based on AKI phenotypes.

Variable	No AKI *n* = 176	Subclinical AKI *n* = 46	Hemodynamic AKI *n* = 17	Severe AKI *n* = 42	*p* Value
Age, years, mean ± SD	63.35 ± 13.4	72.4 ± 13.3	69.4 ± 9.4	74.3 ± 11.	<0.001
Male gender, *n* (%)	30(17)	13(28.3)	3(17.6)	11(26.2)	0.269
BMI, median [IQR]	27.4 [24.7–30.3]	27.9 [25.6–31.3]	27.7 [25.3–29]	25.3 [23.2–28.1]	0.047
Diabetes mellitus, *n* (%)	57 (32.4)	16 (34.8)	5 (29.4)	17 (40.5)	0.763
Hypertension, *n* (%)	92 (521.3)	32 (69.6)	16 (94.1)	33 (78.6)	<0.001
Hyperlipidemia, *n* (%)	103 (58.5)	29 (63)	10 (58.8)	27 (64.3)	0.881
Obesity, *n* (%)	47 (26.7)	15 (32.6)	2 (11.8)	7 (16.7)	0.188
Past MI, *n* (%)	40 (22.7)	19 (41.3)	4 (23.5)	17 (40.5)	0.021
Smoker, *n* (%)	82 (46.6)	22 (47.8)	9 (52.9)	11 (26.2)	0.082
Family history of IHD, *n* (%)	38 (21.6)	3 (6.5)	0 (0)	4 (9.5)	0.008
Heart rate (beats per minute), mean ± SD	77.9 ± 18.6	78.7 ± 16.4	80.8 ± 22.6	89.2 ± 20.3	0.007
Systolic blood pressure (mmHg), mean ± SD	140.9 ± 29.7	145.4 ± 30.7	107.5 ± 8.5	137.6 ± 32.1	<0.001
Diastolic blood pressure (mmHg), mean ± SD	85.6 ± 18.5	82.3 ± 16.9	64.9 ± 7.8	79.4 ± 2.9	<0.001
EF%, mean ± SD	47 ± 8.3	46.5 ± 8.6	42 ± 12.9	41.1 ± 9.1	<0.001
Hemoglobin (g/dL), median [IQR]	14.5 [13.4–15.5]	13.7 [12.8–16.2]	13.6 [12.8–14.5]	12.7 [11.1–15.2]	<0.001
White blood cells (10 × 103/µL), mean ± SD	11 ± 3.9	10.3 ± 3.5	10.5 ± 4.1	12.3 ± 4.2	0.099
Troponin I (ng/L), median [IQR]	23,154 [10,575,72,401]	15,179 [3394–58,105]	17,385 [8471–94,224]	12,707 [738,197,861]	0.683

Baseline characteristics—AKI—acute kidney injury, BMI—body mass index, MI—myocardial infraction, EF—ejection fraction, IHD—ischemic heart disease.

**Table 2:** Multivariate regression for the occurrence of any MACE.

	HR for MACE	95% Confidence Interval		*p*-Value
		Lower	Upper	
No AKI	Reference for baseline hazard			
Subclinical AKI	4.151	2.068	8.331	<0.001
Hemodynamic AKI	4.517	1.608	12.691	0.004
Severe AKI	12.964	5.597	30.028	<0.001
Past MI	0.574	0.308	1.069	0.080

Multivariate regression—multivariate regression was then performed (included AKI phenotype, age, gender, DM, hyperlipidemia, hypertension, family history, smoking, past MI) to calculate HR for MACE when “no AKI” was set as baseline hazard, only independently significant variables are presented. Abbreviations—HR—hazard ratio, MACE—major adverse coronary event, AK—acute kidney injury, MI—myocardial infraction.

## Data Availability

The data presented in this study are available on request from the corresponding author.
